# Silent Sinus Syndrome Post Trauma: A Case Report and Literature Review

**DOI:** 10.7759/cureus.45740

**Published:** 2023-09-21

**Authors:** Sathyanarayanan Ramanujam, C V Srinivedha, Bharathraj Kanniyappan, Deepika Reddy

**Affiliations:** 1 Dentistry Oral and Maxillofacial Surgery, Sri Balaji Vidyapeeth University, Pondicherry, IND

**Keywords:** orbital floor reconstruction, orbit, silent sinus syndrome, enophthalmos, blowout fracture

## Abstract

Silent sinus syndrome (SSS) is a relatively rare clinical condition occurring in the maxillary sinus exhibiting progressive enophthalmos and hypoglobus. The communication between the orbit and maxillary antrum due to trauma further leads to changes in the maxillary sinus. This could lead to the development of negative pressure within the maxillary sinus, collapse of antrum walls, and sucking in of orbital contents. Here, we present a case of a delayed orbital trauma, which was not treated initially. The patient's complaint was a constant feeling of a sunken right eye along with restriction in eye movements. On examination, the clinical and radiographic features were consistent with SSS. Orbital floor reconstruction was performed with the help of a titanium mesh for the correction of hypoglobus and enophthalmos. During follow-up, the patient showed no recurrence of the presenting functional and esthetic complaints. This clinical entity needs prompt diagnosis and early intervention so as to prevent further complications.

## Introduction

Silent sinus syndrome (SSS), also known as imploding maxillary antrum syndrome, involves the asymptomatic and spontaneous collapse of maxillary sinus walls and floor of the orbit [1]. This is a relatively rare condition exhibiting progressive enophthalmos as a result of maxillary sinus atelectasis and retraction resulting in orbital expansion and weakening of the orbital floor. This condition could also lead to progressive hypoglobus [1]. Orbital fractures, especially floor fractures, tend to give rise to enophthalmos. Displacement of orbital contents and surrounding soft tissues into the maxillary sinus through the floor fracture leads to enophthalmos and hypoglobus [2]. Enophthalmos presents after one to two weeks of trauma after the edema and hemorrhage were resolved. There are very few case reports of SSS post orbital trauma [3-4]. We are hereby presenting a case of progressive enophthalmos and hypoglobus post trauma, the findings being consistent with SSS.

## Case presentation

**Figure 1:**
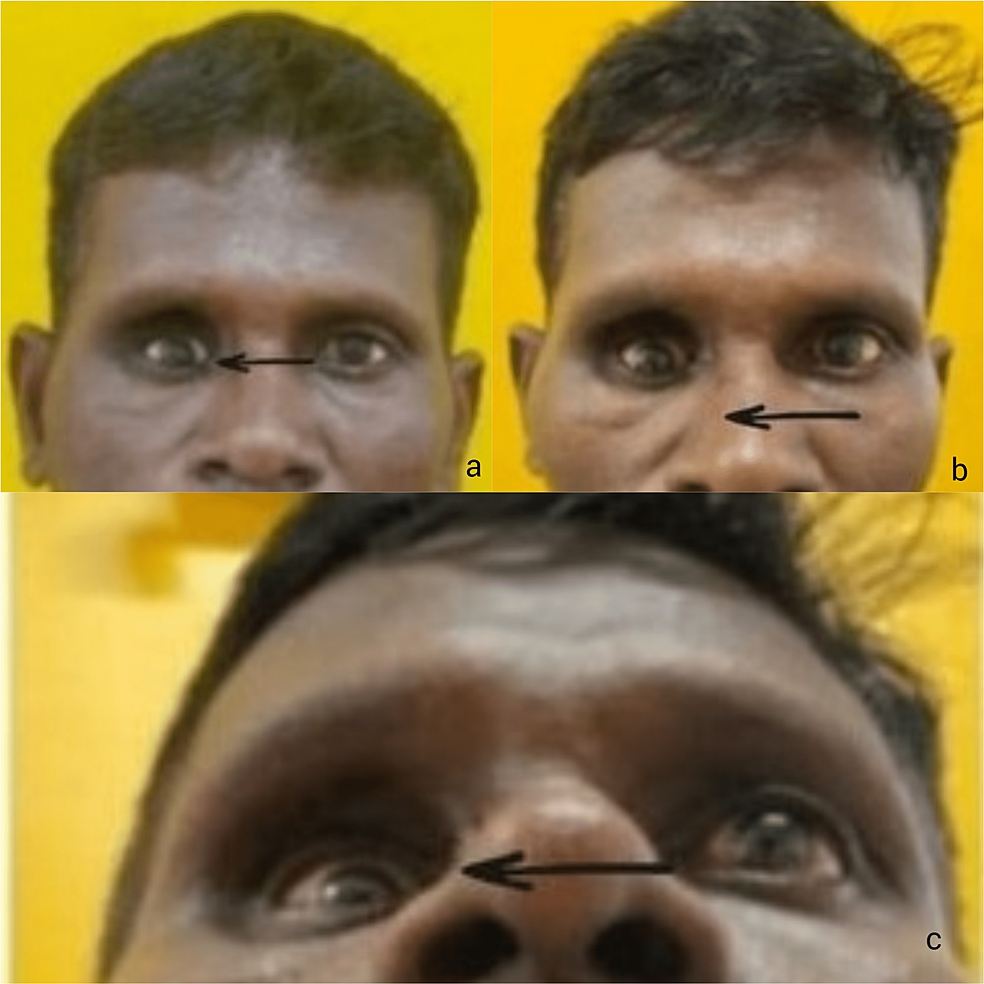
Preoperative photographs of the patient: a) hypoglobus and enophthalmos w.r.t. the right eye; b, c) progressive enophthalmos and hypoglobus w.r.t. the right eye.

A 45-year-old male patient reported to the Outpatient Department of Oral and Maxillofacial Surgery with a chief complaint of pain over the right infraorbital region following trauma that occurred two months back. There was no gross facial asymmetry or palpable step deformity over the right infraorbital region. Right-eye enophthalmos was evident with no signs of diplopia and no loss of visual acuity. Eye movements showed restriction in elevation and dextroelevation, indicative of inferior rectus muscle and inferior oblique impingement. The patient was asked to report with a computed tomography (CT) scan and reports after which the patient had reported two months later with a complaint of a decrease in the eye ball size, unaesthetic appearance, and sunken eye. On examination, there was an increase in right upper eyelid hollowing, hypoglobus, and enophthlamos (Figure 1).

**Figure 2:**
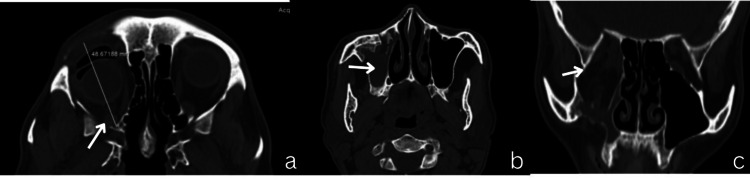
Preoperative computed tomography pictures of the patient. Axial and coronal sections of the CT scan showing enophthalmos, fracture of the infraorbital rim, and retraction of maxillary sinus walls

CT scan revealed fracture of the floor of the orbit, infraorbital rim, and fronto-zygomatic suture region with herniation of the inferior rectus and inferior oblique muscles into the maxillary sinus (Figure 2). 

The right maxillary sinus showed uniform opacity. There was maxillary sinus wall retraction, which ultimately led to a reduction in the volume of the maxillary antrum and orbital floor collapse. ENT opinion was sought as the clinical and radiological features were suggestive of SSS. Ophthalmological examination revealed no abnormalities in visual acuity, and the presence of diplopia was ruled out by Hess charting. The forced duction test was positive. However, an enophthalmos of 4 mm was recorded with the help of the Hertel exophthalmometer. Gaze was restricted in the upper and upper-right lateral directions.

**Figure 3:**
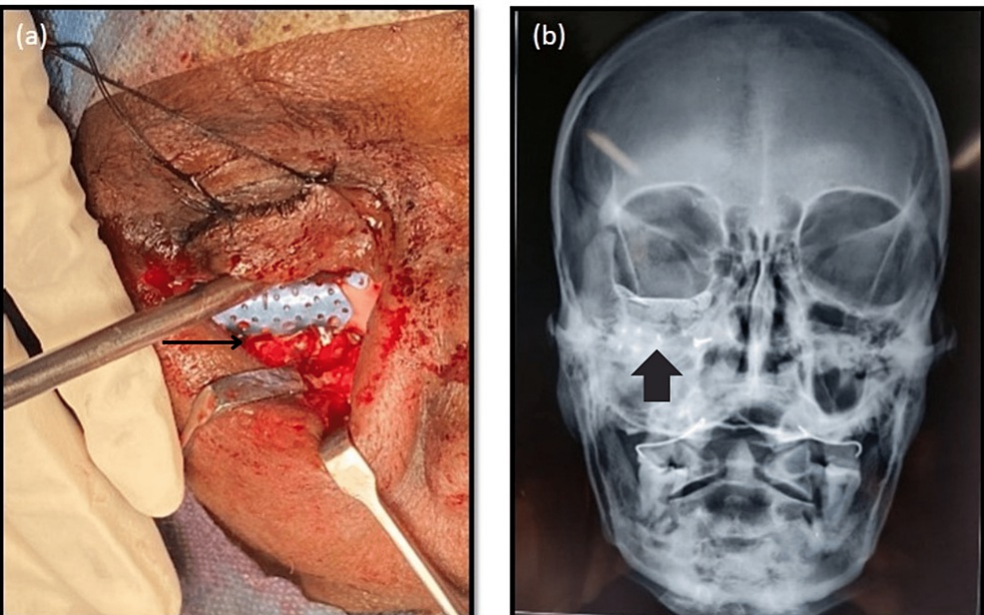
Intraoperative and postoperative pictures of the patient: a) intraoperative: contouring and securing the orbital floor mesh; b) postoperative: X-ray showing titanium mesh placed over the right orbital floor.

The patient’s surgery was planned collaboratively by both the ENT team and oral and maxillofacial surgery (OMFS) team at the same setting. Functional endoscopic sinus surgery (FESS)-assisted middle meatus antrostomy (MMA) followed by orbital floor reconstruction was planned because of a lack of structure in the orbital floor along with the presence of antral obstruction. FESS-assisted MMA was done where the maxillary sinus ostium was widened, and antral wash was given to assist maxillary sinus drainage. The orbital floor was approached through an infraorbital incision, and the herniated inferior rectus and inferior oblique muscles were identified along with fibrous and scarred tissues embedded within the sinus walls entangled with mucin. Re-osteotomy of the infraorbital rim fracture was done to facilitate proper access for retrieving the herniated muscles, scarred tissue was dissected till the posterior palatine ledge of the orbital floor, and the herniated tissues were raised. While relieving the scarred muscles, they were seen to be embedded in mucinous milieu inside the maxillary sinus. Forced duction test was negative after relieving the entrapped muscle. A titanium mesh was then contoured and placed along the orbital floor and secured. Postoperative X-ray was taken (Figure 3).

**Figure 4:**
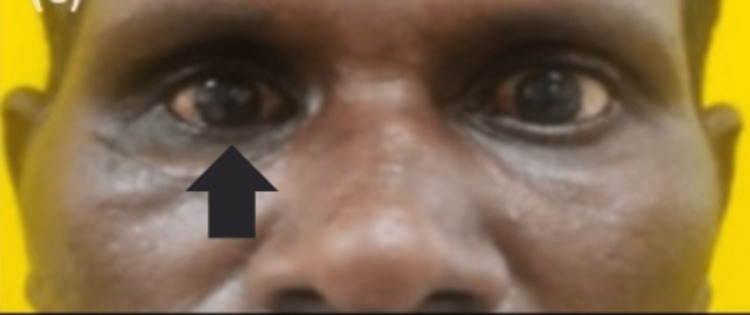
Resolved enophthalmos and hypoglobus w.r.t. the right eye.

The enophthalmos resolved post surgery as depicted in Figure 4 along with improvement in eye ball movements in the upper gaze (since only a straight profile picture is shown without a upper gaze image).

## Discussion

Posterior displacement of the eyeball due to increase in the orbital volume is termed as enophthalmos. Presentation of enophthalmos after orbital trauma is not uncommon post resolution of edema and hemorrhage. Montgomery was the first one to recognize this clinical condition and publish a case report of mucocele leading to enophthalmos [5]. Later, Soparkar described similar cases with the same clinical presentation and coined the term "silent sinus syndrome" for cases of spontaneous enophthalmos and hypoglobus [5]. The probable recommendations proposed for the etiology of this condition by the authors were chronic sinusitis, maxillary sinus occlusion, interruption of normal sinus development, and mucocele or any destructive cysts in the maxillary antrum [5].

Pathophysiology of this phenomenon was proposed by Vander Meer et al. in 2001. Chronic maxillary ostium obstruction will lead to a buildup of negative pressure inside the maxillary sinus, which will consequently pave way to the collapse of the sinus walls and simultaneous sucking in of the orbital floor [5].

Baujat et al. in their case report have explained in detail the effect of masticatory muscles on the maxillary sinus post trauma, thus exhibiting features of SSS [5]. There were few authors who had mentioned about trauma-induced SSS, such as Pawar et al. and Gagnon et al. [5]. Montezuma et al. (2008) presented a case of SSS presenting as enophthalmos [6].

CT findings of SSS were enumerated by Albadr et al., such as inward bowing of maxillary sinus along with maxillary sinus opacity, displacement of the middle turbinate laterally, and implosion of the maxillary sinus, which means retraction of the walls of the maxillary sinus, namely, anterior, posterior, and medial to the lumen of the sinus. MRI complements CT findings, such as sinus opacification, orbital fat prominence, and retroantral fat pad widening [7].

Case reports of post traumatic SSS are relatively less in the literature. We report a case of delayed progressive enophthalmos with clinical and radiological findings consistent with SSS. Through the existing literature, we can enumerate the symptoms of SSS, such as progressive enophthalmos, hypoglobus, preservation of ocular motility with or without gaze restriction, eyelid retraction secondary to lid and globe dystopia, unaffected visual acuity, sinking of the eye or pulling sensation of the eye, orbital asymmetry (superior sulcus deepening), and diplopia (less common, commonly painless with occasional ache in orbital tissues, which is nonspecific).

Repair of the orbital floor using orbital mesh was performed to restore the orbital volume, hypoglobus, and enophthalmos in SSS along with endoscopic sinus surgery [8-9]. Behbehani et al. in their case series of five patients reported that endoscopic antrostomy and porous polyethylene orbital implant placement were done simultaneously, and no adverse outcome was reported [10].

## Conclusions

Orbital fractures constitute an important spectrum of trauma in the maxillofacial arena. While there are many guidelines on the timings to intervene or perform reconstructive surgeries for orbital floor fractures, the decision is solely dependent on the surgeons and their expertise. Increasing incidences of SSS have led to new insights in recognizing the actual changes occurring post orbital floor fractures. Identifying these variations is a need of the hour in developing a proper consensus for the management of such entities and also paves the way for an interdisciplinary approach of patient care.

## References

[1] Yab K, Tajima S, Ohba S (1997). Displacements of eyeball in orbital blowout fractures. Plast Reconstr Surg.

[2] Rosso C, Saibene AM, Felisati G, Pipolo C (2022). Silent sinus syndrome: systematic review and proposal of definition, diagnosis and management. Acta Otorhinolaryngol Ital.

[3] Canzi G, Morganti V, Novelli G, Bozzetti A, Sozzi D (2015). Posttraumatic delayed enophthalmos: analogies with silent sinus syndrome? Case report and literature review. Craniomaxillofac Trauma Reconstr.

[4] Brown SJ, Hardy TG, McNab AA (2017). "Silent sinus syndrome" following orbital trauma: a case series and review of the literature. Ophthalmic Plast Reconstr Surg.

[5] Cobb AR, Murthy R, Cousin GC, El-Rasheed A, Toma A, Uddin J, Manisali M (2012). Silent sinus syndrome. Br J Oral Maxillofac Surg.

[6] Montezuma SR, Gopal H, Savar A, Turalba A, Cestari DM, Torun N (2008). Silent sinus syndrome presenting as enophthalmos long after orbital trauma. J Neuroophthalmol.

[7] Albadr FB (2020). Silent sinus syndrome: interesting computed tomography and magnetic resonance imaging findings. J Clin Imaging Sci.

[8] Sesenna E, Oretti G, Anghinoni ML, Ferri A (2010). Simultaneous management of the enophthalmos and sinus pathology in silent sinus syndrome: a report of three cases. J Craniomaxillofac Surg.

[9] Thomas RD, Graham SM, Carter KD, Nerad JA (2003). Management of the orbital floor in silent sinus syndrome. Am J Rhinol.

[10] Behbehani R, Vacareza N, Bilyk JR, Rubin PA, Pribitkin EA (2006). Simultaneous endoscopic antrostomy and orbital reconstruction in silent sinus syndrome. Orbit.

